# Altered coordination strategies during upright stance and gait in teachers of the Alexander Technique

**DOI:** 10.3389/fragi.2023.1090087

**Published:** 2023-05-04

**Authors:** Molly B. Johnson, Rajal G. Cohen

**Affiliations:** ^1^ Trauma and Injury Research Center, Dell Children’s Medical Center, Austin, TX, United States; ^2^ Kinesiology Department, University of the Incarnate Word, San Antonio, TX, United States; ^3^ Mind in Movement Laboratory, Department of Psychology and Communication, University of Idaho, Moscow, ID, United States

**Keywords:** gait, walking, posture, standing, stance, balance, Alexander Technique, aging

## Abstract

Deterioration in movement and posture often occurs with aging. Yet there may be approaches to movement training that can maintain posture and movement coordination patterns as we age. The Alexander Technique is a non-exercise-based approach that aims to improve everyday movement and posture by increasing awareness and modulating whole-body postural muscle activity. This study assessed whether nineteen 55–72-year-old Alexander Technique teachers showed different posture and movement coordination patterns than twenty age-matched controls during a standing and walking protocol using 3D inertial sensors. During upright stance, Alexander Technique teachers showed lower centroidal sway frequency at the ankle (*p* = .04) and lower normalized jerk at the sternum (*p* = .05) than controls. During gait, Alexander Technique teachers had more symmetrical gait cycles (*p* = .04), more symmetrical arm swing velocity (*p* = .01), greater arm swing velocity (*p* < .01), greater arm swing range of motion (*p* = .02), and lower range of acceleration of the torso in the frontal plane (*p* = .03) than controls. Smoother control of upright posture, more stable torso motion, and less restrained arm mobility suggest that Alexander Technique training may counter movement degradation that is found with aging. Results highlight the important balance between mobility and stability within the torso and limbs.

## 1 Introduction

The control of human movement requires a delicate balance of stability and mobility so ineffective effort can be minimized and postural support and productive actions can be facilitated. During walking, it is thought that trunk motion is stabilized through neuromuscular control (Winter et al., 1993) and that maintaining stability of the head is a priority over limb stability (Cromwell et al., 2004). Research highlights an important link between torso motion and head stability by showing that torso motion can be temporarily degraded in young, healthy people when head orientation is interfered with (Johnson & Van Emmerik, 2010; Johnson & Van Emmerik, 2011). Additionally, potential links between torso stability and limb mobility are highlighted by age-associated degradations. Research shows that with aging, the greatest declines in dynamic stability of the body are in the torso (Kang & Dingwell, 2009). Additionally, increasing asymmetry of gait factors, such as arm swing, is common with aging and is associated with fall risk (Mirelman et al., 2015; Aboutorabi et al., 2016; Gillain et al., 2019).

Balance and movement may improve long-term through therapeutic interventions provided over time. Additionally, balance and movement may show immediate improvement through changes to movement instructions. For example, swinging arms more actively when walking improves gait characteristics in healthy young and middle-aged participants and in people with Parkinson’s Disease (Hu et al., 2012; Weersink et al., 2021). However, movement tips may not offer substantial long-term change for people with functional impairments if they improve one movement pattern without improving other movement patterns or coordination of the whole. Additionally, improvements in one task (e.g., gait) might not translate to improvements in other tasks (e.g., standing balance).

An unconventional approach to postural and movement training called the Alexander Technique may offer useful insights into ways to delay deterioration of movement patterns common with aging. The Alexander Technique is a non-exercise-based approach that aims to improve everyday movement and posture by means of sophisticated modulation of whole-body postural muscle activity. Alexander Technique instruction uses three main principles: 1) improved awareness of the whole body in three-dimensional space; 2) purposeful inhibition of habitual excess muscular activity in the planning, initiating, and carrying out of movement; and 3) the use of mental commands or imagery to establish a more poised and dynamic use of the head, torso, and limbs (Cacciatore et al., 2005). A growing body of research suggests that the Alexander Technique may provide long-term improvements after taking a series of lessons and also immediate improvements using Alexander-based cues, across many posture and movement domains (Dennis, 1999; Stallibrass et al., 2002; Batson & Barker, 2008; Little et al., 2008; Cohen et al., 2015; MacPherson et al., 2015; O’Neill et al., 2015; Hamel et al., 2016; Preece et al., 2016; Loram et al., 2017; Becker et al., 2018; Cohen et al., 2020; Becker et al., 2021). Additionally, research suggests that improvements may translate to other domains and tasks that are not directly addressed by Alexander Technique lessons or classes, such as balance improving without ever having performed balance tasks during lessons (Cacciatore et al., 2020).

Most people seek Alexander Technique lessons to address chronic musculoskeletal pain or to improve posture, general wellbeing, or skilled performance, such as playing a musical instrument (Eldred et al., 2015). Support for the applications to pain and wellbeing is shown by randomized controlled trials demonstrating the effectiveness of the Alexander Technique in reducing back pain and reducing disability in Parkinson’s Disease (Stallibrass et al., 2002; Little et al., 2008). Additionally, research has shown benefits for people with knee osteoarthritis and neck pain (MacPherson et al., 2015; Preece et al., 2016; Becker et al., 2018; Becker et al., 2021). Support for the application to posture and balance is demonstrated by research on quiet upright stance and single-leg stance showing reduced postural sway in older adults and individuals with Parkinson’s Disease when given Alexander Technique-based instructions to think of their upright posture effortlessly compared with conditions where they relaxed or thought of upright posture more effortfully (Cohen et al., 2015; Cohen et al., 2020). Additionally, following a series of Alexander Technique lessons or classes, balance improved in older adults (Dennis, 1999; Batson & Barker, 2008).

Differences in gait patterns have also been associated with the Alexander Technique; during fast walking, older Alexander Technique teachers showed less medial-lateral center of mass displacement and smaller stride width compared to age-matched controls (O’Neill et al., 2015). Additionally, older Alexander Technique teachers showed lower trunk and head motion and greater ankle, knee, and hip motion compared to controls (Hamel et al., 2016). In people with knee osteoarthritis, reduced knee co-contraction was seen during gait following a series of Alexander lessons (Preece et al., 2016). No published research has looked at movement of the arms in people with Alexander Technique training during gait, but it is possible arms would swing more freely, paralleling changes seen in the legs.

The aim of this study was to assess differences in dynamics of upright stance and gait between Alexander Technique teachers and control participants using 3D inertial sensors, to explore whether older Alexander Technique teachers displayed patterns of posture and movement typical of a younger population. During upright stance, we predicted lower sway frequency, sway area, and normalized jerk at sternum, lumbar, and ankles for Alexander Technique teachers compared to controls. During gait, we predicted lower range of motion and acceleration of the trunk, greater arm swing, and more symmetrical limb movements for Alexander Technique teachers compared to controls.

## 2 Materials and methods

### 2.1 Participants and setting

Thirty-nine participants, aged 55–72 years participated in the study. Participants provided informed consent in accordance with the Oregon Health & Science University (OHSU) Institutional Review Board and filled out an intake form that asked about their age, sex, height, weight, and a brief medical history. Participants were eligible for the study if they were 55–75 years old and had no pain on the day of testing, no history of stroke, and no neurological, balance, or orthopedic conditions.

Nineteen of the participants were Alexander Technique teachers (8 male, 11 female). All enrolled Alexander Technique teachers were certified by the American Society for the Alexander Technique (AmSAT) or its international affiliates after completing a 3-year, 1600-h training program, of which 80% was devoted to their own proficiency in the Alexander Technique. Alexander Technique teachers had a mean age of 61.6 ± 5.3 years, a mean height of 168.7 ± 11.0 cm, and a mean weight of 70.3 ± 14.6 kg. Alexander Technique teachers were recruited through an email list of attendees for the annual AmSAT conference. Data were collected in a large, open room at the conference site.

Twenty control participants (6 male, 14 female) were selected from a dataset collected for a larger study in the Portland, Oregon area. Participants were selected based on height, weight, and age, which were used to match the control group to the Alexander Technique teacher group. The mean age of 65.0 ± 5.0 years, height of 166.3 ± 8.0 cm, and weight of 71.7 ± 16.5 kg for the control participants were not significantly different from that of Alexander Technique teachers (*p* > .05). Data were collected in a large, open laboratory in the OHSU School of Medicine.

### 2.2 Experimental procedure

All participants performed three repetitions of the following protocol: 30 s of quiet upright stance, then a 7 m walk, a 180° turn, and another 7 m walk. Control participants were tested by a research assistant trained in the same lab as the second author. Alexander technique teachers were tested by the authors, who were postdoctoral researchers at the time and are trained as Alexander Technique teachers. All participants received the same standardized protocol instructions to start in a comfortable standing position and to walk and turn as they normally would.

### 2.3 Equipment

Data were collected using a portable motion analysis system consisting of six inertial sensors (XSens, Enschede, Netherlands). Each sensor consisted of a 3-dimensional gyroscope (300/s range) and tri-axial accelerometer (1.7 g range). Sensors were attached to the participants’ wrists, ankles, lower lumbar spine, and sternum, and secured using tight elastic wraps and tape. The axes of the sensors were oriented along the anterior-posterior (AP), medial-lateral (ML), and vertical axes. The sensors were serially wired; a cable connected them to a data transmitter on a belt around the waist, which wirelessly streamed the data to a laptop. Acceleration and angular velocity signals were sampled at 50 Hz, transformed to a horizontal-vertical coordinate system, and filtered with a 3.5 Hz cut-off, zero-phase, low-pass Butterworth filter. Gait and balance objective measures were automatically derived from acceleration and angular velocity signals using the APDM Mobility Lab software (APDM, Inc., Portland, OR, United States). Software algorithms automatically separated the different parts of the task and provided separate analyses and measures for upright stance and gait.

### 2.4 Data analysis

During upright stance, sway data were analyzed from the sternum, lumbar, and ankle sensors using three measures: 1) centroidal sway frequency (Hz), calculated using the median power of the acceleration signal; 2) sway area (m^2^/s^5^); and 3) normalized sway jerk, calculated as the derivative of the acceleration signal and normalized to the peak-to-peak acceleration excursion range in the trial (Mancini, et al., 2011). The sway jerk was normalized so it would be less affected by the amount of sway and more an indicator of the smoothness and the degree of regulatory postural corrections (Bottaro et al., 2005). Normalization made the jerk variable unit-less.

During gait, we compared cadence (steps/min), stride length (% height), and stride velocity (% height/s) between groups to ensure there were no general differences that might affect other variables. We also assessed the double support time (% of gait cycle), and gait cycle asymmetry (% difference in stance phase between left and right sides). Additionally, the arm range of motion in the pitch direction (°), peak arm swing velocity (°/s), and arm swing velocity asymmetry (% difference between left and right sides) were assessed. For the trunk, range of motion (°) and range of acceleration (m^2^/s^2^) in the frontal plane were assessed. Differences between groups were determined using independent t-tests with *α* ≤ .05.

## 3 Results

### 3.1 Upright stance

**FIGURE 1:**
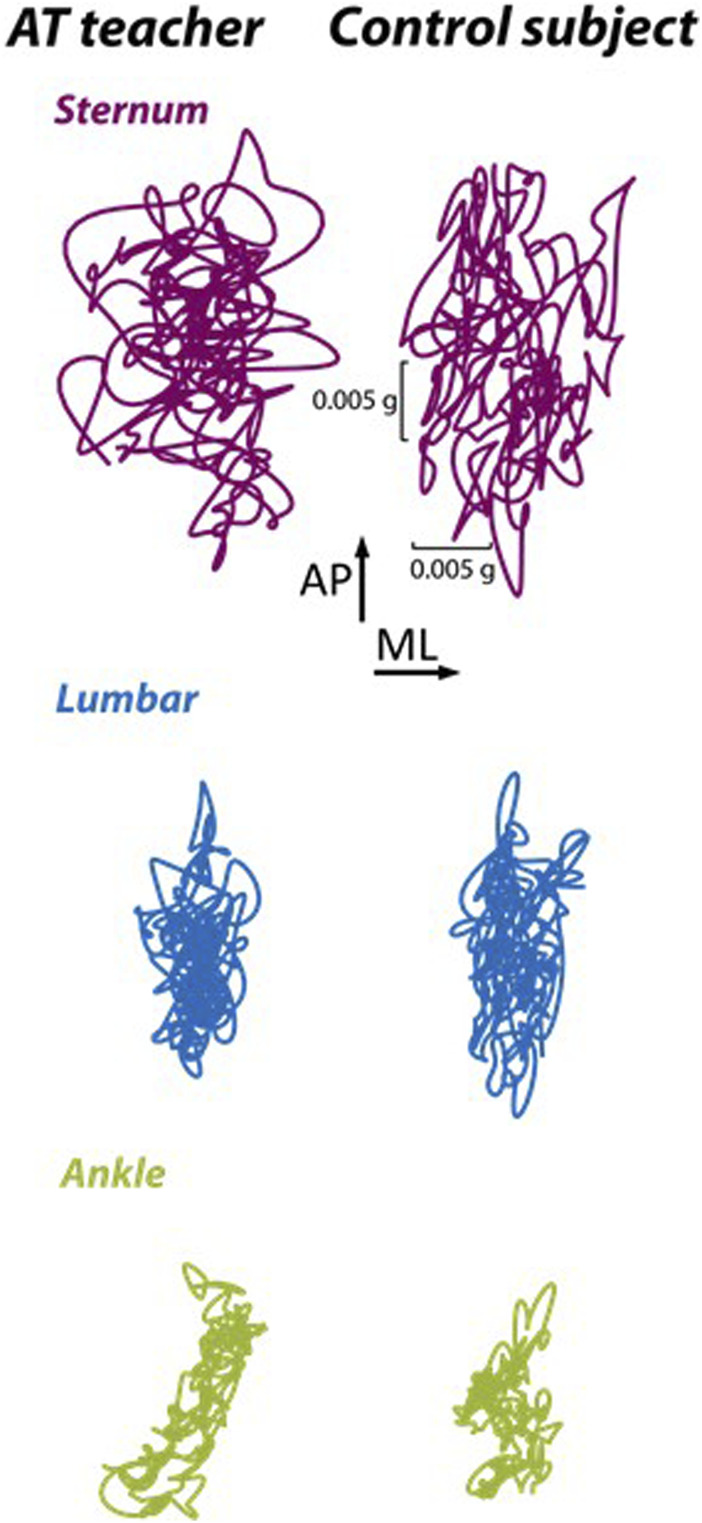
Representative sway trajectory during 30 s of upright stance from the lumbar sensor of an Alexander Technique (AT) teacher and a control group participant (Control) shown within the anterior-posterior (AP) and medial-lateral (ML) axes.

Centroidal sway frequency was 14.8% lower for the Alexander Technique teachers than for the control group at the ankle (*p* = .04) but was not different at the lumbar spine (*p* = .49) (Table 1). Centroidal sway frequency was non-significantly lower for the Alexander Technique teachers than for the control group at the sternum (*p* = .09). There were no significant differences in sway area at the ankle (*p* = .11), lumbar spine (*p* = .34), or sternum (*p* = .21). There were no significant differences in normalized jerk at the ankle (*p* = .19) or lumbar spine (*p* = .22). Normalized jerk was 11.4% lower in Alexander Technique teachers than in controls at the sternum (*p* = .05). See Figure 1 for representative sway patterns at each segment.

**TABLE 1:** Comparison of upright stance characteristics between Alexander Technique teachers and a control group.

Outcome measure	Location	AT	Control	*p*-value
Centroidal sway frequency (Hz)	Ankle	0.654	0.768	.04
	Lumbar Spine	0.777	0.779	.49
	Sternum	0.754	0.821	.09
Sway area (m^2^/s^5^)	Ankle	0.003	0.002	.11
	Lumbar Spine	0.003	0.003	.34
	Sternum	0.005	0.006	.21
Normalized jerk	Ankle	3.883	4.250	.19
	Lumbar Spine	5.271	4.902	.22
	Sternum	4.439	5.012	.05

Abbreviations: Alexander Technique teachers (AT), hertz (Hz), meters (m), second (s).

### 3.2 Gait

There were no significant differences in cadence (*p* = .10), stride length (*p* = .08), or stride velocity (*p* = .08) between Alexander Technique teachers and the control group (Table 2). There were no significant differences in the percentage of the gait cycle spent in double support (*p* = .10). However, the time spent in stance was 29.3% more asymmetrical between the two legs for the control group than for the Alexander Technique teachers (*p* = .04).

**TABLE 2:** Comparison of gait characteristics between Alexander Technique teachers and a control group.

Outcome measure	AT	Control	*p-*value
Cadence (steps/min)	111.843	115.408	.10
Stride length (% height)	85.806	87.679	.08
Stride velocity (% height/s)	80.024	84.366	.08
Double support time (%)	24.721	22.691	.10
Gait cycle asymmetry (%)	3.749	4.849	.04
Arm swing range of motion—pitch (°)	21.332	14.169	.01
Peak arm swing velocity (°/s)	173.811	131.766	<.01
Peak arm swing velocity asymmetry (%)	18.514	26.656	.02
Torso range of acceleration—frontal plane (m^2^/s^2^)	0.874	1.006	.03
Torso range of motion—frontal plane (°)	3.771	4.422	.10

Abbreviations: Alexander Technique teachers (AT), minute (min), second (s).

Alexander Technique teachers had 50.6% greater arm swing range of motion (*p* = .01) and 31.9% greater arms swing peak velocity (*p* < .01) than the control group. Additionally, the control group showed 44.0% more asymmetric arm swing velocity than Alexander Technique teachers (*p* = .02).

In the frontal plane, the range of acceleration of the torso was 13.1% lower in Alexander Technique teachers than in the control group (*p* = .03). Torso range of motion was lower in Alexander Technique teachers than the control group, but this difference did not reach statistical significance (*p* = .10).

## 4 Discussion

### 4.1 Summary of results

This study found that during upright stance, Alexander Technique teachers showed lower centroidal sway frequency at the ankle and lower normalized jerk at the sternum than the control group. During gait, Alexander Technique teachers had more symmetrical gait cycles, more symmetrical arm swing velocity, greater arm swing range of motion and velocity, and lower range of acceleration of the torso in the frontal plane than the control group.

### 4.2 Interpretation: upright stance

During upright stance, the lower centroidal sway frequency at the ankle and lower normalized jerk at the sternum suggest smoother control of body segments in the regulation of upright posture in Alexander Technique teachers compared to the control group. This strategy could be optimizing the coordination of multiple body segments in the control of upright posture to minimize acceleration of the center of mass (Aramaki et al., 2000).

### 4.3 Interpretation: gait symmetry

During gait, our results highlight greater symmetry in both the arms and legs for Alexander Technique teachers compared to the control group. Generally, more symmetrical gait patterns are associated with healthier movement. Research shows that symmetry of arm swing and other gait characteristics decreases with age (Mirelman et al., 2015; Aboutorabi et al., 2016). In older adults, asymmetry during walking when a dual task was present was associated with an increased likelihood of falling (Gillain, 2019). Our findings highlight that Alexander Technique teachers use more symmetrical gait strategies associated with a younger population and reduced risk of aging-related falls.

### 4.4 Interpretation: gait—torso and limb motion

Arm swing and trunk motion are related aspects of locomotion. During gait, increasing arm swing increases trunk stability more in older compared with younger adults (Hu et al., 2012). The differences in gait characteristics seen in Alexander Technique teachers are not likely driven by intentionally changing arm movement, though. When people apply the Alexander Technique to movement, they generally prioritize how they are using the head and trunk while de-prioritizing direct control of the limbs (Cacciatore, Horak & Henry, 2005). This approach could stabilize the torso while allowing for a release of restrictiveness in the motion of the limbs. Our research supports prior findings of reduced motion of and within the torso during gait for Alexander Technique teachers, combined with greater range of motion in legs (Hamel et al., 2016). This study takes a novel approach to also show increased arm swing in Alexander Technique teachers. During gait, lower range of motion of the arms and increased trunk accelerations in the control group may suggest age-related gait deteriorations (Kang & Dingwell, 2009; Mirelman et al., 2015), whereas the greater arm swing motion and velocity and decreased trunk acceleration of older Alexander Technique teachers may signify that they are maintaining more youthful movement patterns.

### 4.5 Proposed mechanisms

Prior research shows that across multiple movement domains, the Alexander Technique changes overall coordination in a consistent manner, including reduced torso motion, increased limb mobility, and smoother posture and movement strategies (Cacciatore et al., 2011a; Cacciatore et al., 2014; O’Neill et al., 2015; Hamel et al., 2016). This pattern has been seen across tasks including upright posture, gait, and movement transitions. For example, during the transition from sitting to standing, Alexander Technique teachers use less spinal flexion and extension, lower center of mass velocity, and a smoother weight shift onto the feet than a control group (Cacciatore et al., 2011; Cacciatore et al., 2014). During this movement, Alexander Technique teachers demonstrate the ability to smoothly perform slow movements that people without Alexander Technique training are incapable of performing even when carefully instructed, suggesting that the Alexander technique facilitates reduced trunk and hip stiffness and improvements in dynamic modulation of postural tone (Cacciatore et al., 2014). More generally, it has been proposed that the changes in coordination and reductions in pain associated with practice of the Alexander Technique are due to improvements in the adaptability and distribution of postural muscle tone and refinements in body schema (Cacciatore, Johnson, & Cohen, 2020).

### 4.6 Strengths and limitations

A strength of this study is that our findings offer conceptual replication of results from other labs that used different measurements and different movements. A limitation is that differences in the data collection setting between groups could have affected behavior. Another limitation of this cross-sectional study is that Alexander Technique teachers may differ from the control group in some unknown way that affects the results. For example, Alexander Technique teachers may be more likely to have a background in the performing arts or may have chosen to pursue the Alexander Technique as a way or resolving pain or movement issues that were less common in the control group (Eldred et al., 2015). The coordination differences illuminated here are unlikely to be due to differences in physical fitness. Alexander Technique teachers had similar BMIs and walking speeds compared to the control group. In addition, results of a recent intervention study indicated that reductions in neck pain following Alexander Technique classes were due to different mechanisms than reductions in pain following exercise classes (Becker et al., 2021). Potentially different mechanisms for reducing pain may support additive effects of Alexander Technique lessons and exercise, as was found in a clinical trial for people with low back pain (Little et al., 2008). Additionally, the Alexander Technique can be used by people who may not be able to exercise or who have posture or mobility limitations. No special equipment is needed, so the Alexander Technique can be delivered in any setting. Although often taught privately, research supports the effectiveness of group lessons, making the work both accessible and scalable (Becker et al., 2021).

## 5 Conclusion

Results of this study suggest that the coordination patterns of older Alexander Technique teachers are different from those of an age-matched control group. During upright stance, Alexander Technique teachers showed smoother postural sway than the control group. During gait, they showed greater symmetry of limb motion, greater arm swing, and reduced trunk motion compared to the control group. These changes in coordination suggest that long-term practice of the Alexander Technique may slow some of the deterioration in postural control and gait typically associated with aging. Future research should continue to explore changes in posture and movement associated with the Alexander Technique and further explore the mechanisms driving the changes.

## Data Availability

The raw data supporting the conclusion of this article will be made available by the authors, without undue reservation.
